# Hospital and Institutional News

**Published:** 1912-02-03

**Authors:** 


### HOSPITAL AND INSTITUTIONAL NEWS.

### THE DUKE OF FIFE AND HOSPITAL WORK.

The death of the Duke of Fife removes a member of the Court whose interest in hospitals was a very practical one. The Duke was a member of the General Council of King Edward's Hospital Fund and President of the Great Ormond Street Hospital for Sick Children, and also a Vice-President of the Royal Hospital for Incurables, Putney Heath.

### THE CHARITY COMMISSIONERS AND SPALDING HOSPITAL.

It is announced that the Charity Commissioners have informed the trustees of the Johnson Hospital, Spalding, that they propose to hold an inquiry open to the hospital authorities and other parties interested. As we go to press the date of the investigation is not yet known, but the interval already is being spent locally in much comment, which assumes, what is obviously the case, that the reported action of the Charity Commissioners is due to the effect of Sir Henry Burdett's Reports in recent issues of The Hospital. We regret to note that Canon Bullock, the Chairman of this Hospital, is reported to have again stated, at the annual meeting on 29th inst., that Sir Henry was in the hospital five minutes before the Trustees met, but declined to wait and see them. This was shown in all the local papers weeks ago to be contrary to the facts.

### WHAT THE COMMISSIONERS CAN DO.

The Charity Commissioners are, chief Mr. Charles A. Cook, C.B., Mr. A. F. Leach, and Mr. C. P. Allen. It would be as improper as it is unnecessary to forecast any line of action which they may decide to take, but it is safe to say that should they decide to increase the representative nature of the trust, that result would be perfectly within their competence. Something like a local panic apparently has ensued, as may be guessed from such wild reports as this, that the Commissioners would be unable to vary the trust to a great extent because of its relatively recent foundation. On the contrary, it may be pointed out, the tendency of recent legislation has been largely to increase the powers of the Commissioners and to concentrate their effectiveness of action by transferring all otherwise assignable endowments—educational trusts, for instance—to special departments cognate to them.

### OUR COMMISSIONER'S VISIT TO BEDFORD.

Readers of the illustrated interview which the recently appointed secretary of the Bedford County Hospital, Mr. Beauchamp Wadmore, courteously gave to our commissioner the other day, will note that there are two claimants to any surplus the yearly accounts may show. The first of these is Mr. Tilling, the house surgeon, who, supported by the medical staff, is exceedingly anxious that proper space, accommodation, and up-to-date fittings shall be given to the present cramped *x*-ray room. In Mr. Tilling's words, "improvement is emphatically needed." The second claimant is a section of the board of management, which, judging from the interview, is anxious to replace the old hand-laundry with a machine-laundry of some kind. Mr. Wadmore, it should be noted, is not wholly in favour of this. Some new laundry appliances he does hope for, but he will have the support of the majority of experienced secretaries in his statement that no laundry could be run much more cheaply than the hand-laundry now in use. Hand labour is cheapest in the end, and often more satisfactory to the hospital's linen. From contrasting the proposals of the two claimants for improvements, as we pointedly contrast them in this note, it will be felt that probably the board of management would be very wise to follow the medical staff's advice by modernising the *x*-ray department, leaving the hand-laundry for the present with the minor improvements it needs.

### NATIONAL INSURANCE DEVELOPMENTS.

The profound changes which the Insurance Act is likely, in our judgment, to bring about in the work and system of the voluntary hospitals as hitherto carried on have from week to week received attention in these columns. The medical profession is so intimately concerned in hospital administration that anything which affects the latter must affect the former too, and the doctors have, in fact, been among the first to foresee how seriously the Act affects hospital prospects and principles. So, too, conversely, everything which affects the medical profession must receive the sympathetic consideration of hospital workers beyond all other sections of the public. If hitherto The Hospital has seemed to devote its attention too exclusively to the hospital aspects of National Insurance, it has been very largely because the cause of the doctors has been so ably championed in the leading medical journal. The moderate, reasonable tone in which this question has been handled in the pages of the *Lancet* has been no less remarkable than the statesmanlike outlook and the logical thinking which have distinguished the utterances of the editor, and it is with genuine pleasure and admiration that we acknowledge those qualities.

### WHAT SCOTLAND IS DOING.

In Scotland they have formed a Central Medical Insurance Council, under the auspices of the Royal College of Surgeons of Edinburgh. The published list of delegates shows that this Council is a very large body, perhaps too large to cope successfully with the work involved—namely, the upholding of the dignity of the medical profession in Scotland and of the best interests of the assured. The *Lancet* wishes to see a similar line of action initiated in England by the Royal Colleges, and argues forcibly the merits of such a proceeding. Upon the formation of such a Central Council, the editor believes, a number of persons, now dissenting violently among themselves about details, would be prepared to make a common stand against exploitation of the medical profession, while desirous of associating that profession with any great measure for the advancement of health and the alleviation of misery in this country. Unfortunately, the constitution of the Royal Colleges is not democratic enough to keep their Councils well disposed towards new and up-to-date reforms; and it is to be noted with regret that the Royal College of Physicians has declined an invitation to send two members to a conference called by the Insurance Commissioners of England, Scotland, and Wales. It is to be hoped that the able advocacy of *The Lancet* will persuade both the Colleges to take a different action in respect to the suggestion that England should imitate Scotland in this matter. Meanwhile we may note that the Insurance Commissioners are in touch with the Reform Committee of the British Medical Association, a fact which shows that the authorities are beginning to realise that the profession is in earnest and must be taken seriously. This is all to the good, and it only shows that with a bold front, a spirit of unity, a clear notion of grievances and of the necessary remedies for them, the hospitals also may get justice done; but only by working hard and unceasingly, and only by impressing on public opinion the grave danger with which the Act as it stands threatens the whole of the present system of the voluntary hospitals.

### AN IRISH HOSPITAL ON THE INSURANCE ACT.

Mr. R. B. Walkington, the honorary secretary of the Belfast Hospital for Sick Children, last week presented an annual report to the supporters of his hospital, which animadverts critically upon the National Insurance Bill. The works collection, it is feared, will suffer, and the Board points out that "there is no insurance and no benefit for the children when hurt or ill," and "warns the public that should the shrinkage of funds continue the position of the hospital will be endangered." The hospital's president, Captain the Hon. Arthur O'Neill, M.P., went so far as to say that possibly the Act "would affect children's hospitals like theirs even more seriously than in the case of institutions which dealt with grown-up patients." He might have gone further and said that where one class of voluntary institutions is seriously threatened the whole voluntary system is bound to suffer in the end. As regards local hospital matters, it should be remarked that Mr. Arthur Atkinson, formerly accountant, was recently appointed to fill the vacancy created by the sudden death of Mr. A. A. Shaw, the late assistant secretary.

### A BOOK ON THE INSURANCE ACT.

An exposition of the National Insurance Act at the popular price of one shilling is surely, at the present time, one of those books which may be truthfully said to be really timeous. Nothing less than a ponderous legal treatise will be necessary for those who require to get to the bottom of the complexities of the Act, but as a guide of a popular nature Mr. Thomas Smith's "Everybody's Guide to the Insurance Act, 1911" (Charles Knight and Co., Ltd.), will carry the reader far on his voyage of inquiry. The circumstances under which the Act received its final shape have rendered the author's task a difficult one. To keep pace with the rapid succession of amendments which has characterised this measure has caused, as the author confesses, "both confusion and irritation of mind." However, the result is satisfactory as far as it goes, and we can assure our readers that this guide is well worth consideration by institutional secretaries, householders, and all employers. As an introduction to this now pressing problem we do not think it can be beaten. Although inconveniently bound, the pages are clearly printed and the text is lucid enough. A full reprint of the Act itself and a very useful index are included in its three hundred pages. A good book is always cheap at a shilling.

### THE EXHIBITION OF OPEN-AIR SHELTERS.

The interest which The Hospital awakened and has nourished in the complex question of the designing of open-air shelters has culminated in the exhibition of shelter plans and models now on view at the Royal Sanitary Institute, Buckingham Palace Road. Among the first visitors were Mr. John Burns, and the Medical Officer to the Local Government Board, Dr. A. Newsholme, M.D., C.B. Our own plan, that of publishing the actual shelters that sanatoria and hospitals have designed to meet local needs and specific problems, has proved exceedingly instructive, and from the correspondence it has induced has shown how infinitely more useful to superintendents and architects is the publication of a series of plans now embodied than the exposition of theories however ingenious. One of the most interesting lessons these plans have taught has been that practically no institution requires a shelter for the same purpose as any other and that, in consequence, the shelters made or designed on the premises of the institutions which required them have proved more successful, and also cheaper, than the majority of those supplied by public firms. A walk through the present exhibition confirms this impression.

### A HUMOURIST AND A HOSPITAL.

It is many a year now since *Punch* made its celebrated and successful appeal to the public on behalf of the Hospital for Sick Children, Great Ormond Street. The event, however, is recalled by the act of one of the most brilliant and ingenious authors that ever filled "Mr. Punch's" pages with fantasy. We refer to the author of that evergreen classic, *Vice Versâ*, Mr. F. Anstey, who has written a book entitled *Winnie*, and is publishing it for the benefit of the same hospital, whence copies may be obtained for the sum of half a crown. The story is announced as both pathetic and amusing; and, indeed, the author is one whom one knows of old as capable of provoking that mirth which is akin to tears. This association of author and hospital is one upon which we would beg leave to congratulate both. We trust that the sale of the book is proving rapid and profitable, though we feel obliged to add that it has not been given that publicity which would be at once the truest compliment to the author and the best method of augmenting the resources of the charity.

### THE PAY OF WOMEN TYPISTS IN HOSPITALS.

The letter we publish this week signed " Hospital Secretary " raises a very debatable point. Taking the instance he quotes, the question resolves itself into the general one: is thirty-six shillings a fair wage for a woman's skilled work during a forty-one-and-a-half-hour week? This assumes that the lady has a fortnight's holiday in the year during which she is paid nothing. The effect is thus slightly to heighten the average weekly rate, though the total for the year remains the same. Thus stated, " Hospital Secretary's " senior woman shorthand-typist is paid at the rate of rather less than one shilling per hour for her skilled work. As wages go, we are glad that her pay is as much as our informant states, but as wages ought to be it is obvious that the sum is inadequate. A " living " that is in practice a subsistence-wage in London has been reckoned, we believe, at approximately eighteen shillings a week. Our Pecksniffs and Chadbands will say at once that twice the subsistence-wage is a generous margin, forgetting that woman typists, in spite of all the political economists, are generally middle-class women, and as such their subsistence-wage is considerably higher than the absolutely theoretical figure, which applies to what the working labourer can earn. What happens in practice is, no doubt, that many woman typists still live at home or with relatives of some sort, and thus pay little or nothing for rent and board. Have the hospitals, of all employers, the right to exploit this dependence and to cut down expenses by taxing these humble family tills? To cut them down to subsistence level may be academic political economy. The student of vital economy knows that grievous social penalties result from letting wages fall, as they do among unorganised labour, to their theoretically subsistence level. We do not allude to what will be called the treasurer's point of view, because we have never believed that a hospital does anything but suffer from the underpayment of any member of its staff, though in the present case the wage of the expert typist alone has been given. If any of our readers can prove that we are wrong in this view, we shall be glad to publish the most trenchant arguments that they can advance.

### A CLERGYMAN'S ODD SUGGESTION.

At the Leeds Maternity Hospital's annual meeting the other day the Rev. D. M. M. Bartlett created much laughter by suggesting that a notice of the work of the hospital should be sent to all people who announced births in the columns of the county paper.

### A RETIRING LIVERPOOL CHAIRMAN.

Mr. H. Wade Deacon, who is now retiring from the post of chairman of the committee of the David Lewis Northern Hospital, Liverpool, has received a eulogy from Lord Derby, the president. Lord Derby said how much Mr. Wade Deacon's efforts on the hospital's behalf were appreciated, and how much the severance of his official connection with the hospital is regretted. They all conveyed to him their most grateful thanks for his great services. Mr. Wade Deacon has held the office of chairman for ten years, and has been treasurer for seven; therefore the public can appreciate the sincerity which inspired the praise that has been given for his hospital record. It is further stated that Mr. Wade Deacon has been offered the chairmanship of the Royal Infirmary, Liverpool. Let us add, by the way, that the appeal started by Lord Derby after the mill explosion which The Hospital recorded some weeks ago has realised £3,436.

### TWO DEATHS AMONG HOSPITAL SECRETARIES.

We have to publish this week, by a sad coincidence, obituary memoirs of two hospital secretaries, each of whom has done exceedingly useful work in his time: the late Mr. Arthur Edward Reade, formerly secretary of Charing Cross Hospital, whose funeral took place on Monday last, and the late Mr. Samuel Welsh, founder and formerly secretary of Walsall Hospital. One was a London, the other a provincial officer. Both were men who interested themselves in many sides of social work besides that which belonged to their hospital life. Their records show what a wonderful education, in the statesman's sense, hospital life is to those who are capable of its opportunities. The readiness with which other social work came to them shows how many-sided are the qualities required in, and produced by, a long direction of a hospital's secretarial office.

### THE OTHER SIDE OF THE ANTI-PHTHISIS CAMPAIGN.

Wales especially seems to be enthusiastic over what is called the anti-phthisis campaign, and the latest step appears to have been taken by the Cardiff Guardians. Lord Bute's offer to sell them twenty-five acres of land at Llandough at a cost of £5,000 has been accepted, and a sanatorium for consumptives will be built. Hitherto, apparently, some considerable expense has been incurred in sending phthisical cases from among the 1,300 inmates of the Guardians' two workhouses to sanatoria at distant places. Mr. A. J. Harris, clerk to the Guardians, believes therefore that building a hospital will prove economical in the long run. The interesting point to the general public is that this decision has been arrived at almost simultaneously with the announcement that the fund, now to be called the King Edward Welsh Memorial Association, amounts to £200,000. Apart from this the Welsh Insurance Commissioners, in the words of Mr. David Davies, M.P., have made Wales " a unit for fighting tuberculosis." If the commissioners, the King Edward Welsh Memorial Fund, and finally local authorities are all to cover the Principality with their own sanatoria is there not a risk of overlapping, wasteful expenditure and want of co-ordinated control?

### NEW PRESIDENT AT SOUTHAMPTON INFIRMARY.

The Southampton Guardians have been recommended by their Infirmary Committee to promote Mr. J. C. Young to be resident medical superintendent, on the resignation of his previous chief, Dr. Russell Bencraft. Consequently, the recommendation will probably mean that an assistant resident medical officer is advertised for. Mr. Young's previous appointments include that of house surgeon to the Birmingham General Hospital, and assistant resident medical officer to the Birmingham Workhouse Infirmary.

### THE COMING EXTENSION TO ROCHDALE INFIRMARY.

Mr. T. Elvyn Kershaw, secretary of the Rochdale Infirmary, is naturally grateful to the working people in his neighbourhood, for not only has their help converted a debt of £128 into a balance in hand of £169, but £207 have been raised on their part towards the coming extension. The assessor, Mr. Alex. Graham, F.R.I.B.A., has been considering his award, and we are interested to learn that the architect chosen is Mr. Hugh Healey, of the firm of Jesse, Horsfall and Healey, Rochdale. The terms of the competition did not bind the Building Committee, which will now meet to accept the award. A new ward, with, we understand, the rather large number of twenty-four beds, and a new theatre are to form the most important parts of the extension scheme. At this important juncture Dr. Kerr, J.P., is again president, Mr. W. Davidson honorary secretary, and Mr. W. J. Petrie, J.P., has been appointed a new member on the committee, to fill the vacancy caused by Mr. F. A. Verity's resignation. Last year was memorable, if only for the raising of £28,500; this year will begin the responsible work of carrying the extension into effect.

### FINANCIAL POINTS FROM CHICHESTER INFIRMARY.

Last week we drew attention to the financial improvement that has taken place in the old war between debit and credit at Chichester General Infirmary. In the interval more detailed information has reached us, the effect of which is to drive home the well-known lesson that comparisons between one financial year and another are not as easy as even experts sometimes fancy. The year 1910, for the purposes of Chichester Infirmary, for example, contained fifteen months, so as to enable the hospital to alter its financial year from October 1 to January 1. Again, and more important, in 1900 the uniform system of accounts had not been adopted. Other points of interest are the astonishing increase in the fifteen months of the sums collected in the boxes, which jumped up actually £200—from £66 to £267.

### THE INTELLIGENT HANDLING OF FIGURES.

The great success achieved in increasing the annual subscriptions we alluded to last week. The outstanding fact which epitomises this improvement, and for which we have direct confirmation, is that 1911 was the first occasion the institution paid its way, and without the aid of legacies. Figures are often supposed to make dull reading: the present examples show how interesting figures can be when handled intelligently. For this we take only such credit as is due to the means which secures expert co-operation. The annual meeting on February 8 promises, in the light of this, to be of first-rate interest, and the conduct of that meeting and the attention aroused by it will be the final test of the success of the present management. With this hint we wish Mr. Harrison, the secretary, another successful year, and a report as valuable as that just issued by the Leicester Infirmary.

### AN IRONICAL COTTAGE HOSPITAL.

Cottage hospital reports are not often witty, but the exquisite irony of the following statement in the Budleigh Salterton Cottage Hospital report deserves quoting: "The Committee thank most warmly the clergy who contributed offerings from their several churches, but they regret that the total amount received gets smaller year by year." Direct appeals to the local clergy evidently have fallen on deaf ears, and an attempt is now made gently to shame them into activity. The Committee further adds constructive criticism of a sort, suggesting that "one Sunday in July or August should be set aside as Hospital Sunday." The clergy in the early days of this movement, which their supineness and want of enthusiasm is gradually letting slide down the hill of their inertia, did not need to be reminded that their responsibility only begins with their undertaking to preach a sermon. Hospital Sunday sermons used to be the best in the year. Now they are often perfunctory and mere lip-service. The Sunday movement is too great to be destroyed by little men, and whoever put on record the above stinging sentence has begun to do good service to his hospital. If it were Mr. R. W. Friend, the secretary, we hope he will press his statement home till the clergy awake to do their best for the cause which their predecessors in the pulpits once made the most kindling appeal of the year.

### SHOULD A RESIDENT DISPENSARY STAFF BE WHOLE TIMERS?

Few dispensary secretaries can ever have presented a more satisfactory account of a year's work than Mr. R. H. Lynn, secretary of the Lincoln General Dispensary, has been able to do. It was unfortunate that the actual drafter, Mr. R. J. Ward, was prevented from being present on the occasion, but his satisfaction no doubt lay in the knowledge that after an absence of legacies in 1910 the fine total of £1,000 has been received in the past twelve months, while one factor in the expenses has been cut down from £450 to £300 by dispensing with the second house-surgeon, thus leaving Dr. A. E. Martin in sole charge. No seconder was found for Mr. G. S. R. Lowe's proposal to allow the house-surgeon to act as locum to a neighbouring practitioner in so far as that would not interfere with his dispensary duties, and Mr. Brooks hinted that if it had been adopted the subscribers might think the house-surgeon's salary too high, and subscribe less by way of disapproval, "as it was a charitable work." We cannot enter again on the whole question of the payment of medical staffs and its desirability, but we would point out that the question is really whether because a man is paid a good salary—as salaries go—his whole time should be demanded. State hospitals most certainly would so demand it, the point is of course that successful practitioners who are now glad to keep their hands in by hospital work, would ask the State, if their whole time were demanded (as it would be), for a salary proportioned to their success in practice, and that would make their demands prohibitive, and probably an inferior type of practitioner would take their place.

### A HOSPITAL TURNED INTO A LABOUR EXCHANGE.

The new cottage hospital at Goole, to be known as the Bartholomew Hospital, is expected to be finished by May Day. Mr. Henry Chapman, in referring to this at the annual meeting, remarked that the present building, now inadequate for hospital purposes in the district, will then immediately be let as a labour exchange. The rent derived from this purpose will, he said, go a long way towards maintaining the new hospital. The fate of buildings that were once hospitals is now growing as curious as the sort of buildings chosen forty years ago for hospital adaptation. The present cottage hospital, now being abandoned, at Axminster was originally the famous factory for Axminster carpets. The present building at Goole, now equipped as a hospital, may perhaps become famous as a labour exchange: such are the topsy-turveydoms of architecture.

### "THE HOSPITAL" AND ANOTHER PAPER.

After our comment last week upon a type of criticism which was being levelled at Sir Henry Burdett's recent report upon the condition of the Hull Royal Infirmary, there is some relief to be gained by the perusal of a paragraph which appeared in the "Yorkshire Observer" on Monday. Under the heading "Yorkshire Hospitals and Efficiency," attention is drawn to Sir Henry's just-published report upon the York County Hospital, and the writer concludes: "Anyone who has read Sir Henry Burdett's reports cannot but be struck by their directness; and although in some cases they do not find favour, it is probable that in others substantial improvements have been effected in the condition of hospitals and infirmaries through the advice and criticism tendered by such an expert." This, in spite of its caution, makes it evident that this writer has paid some attention to the reports which have appeared in our pages from time to time, and equally evident that he has grasped the spirit of the suggestions made in them. Incidentally the observation concerning the directness of the remarks adds weight to our statement last week that the reports are nothing but recitals of fact. We are content to let the facts speak for themselves.

### LEEDS GUARDIANS AND A WIDOW'S MITE.

We hope that the Leeds Board of Guardians have some better reason for refusing relief to the widow Mary Quick and her three children than appears from the full report in the "Leeds Citizen" of their dealing with the case. Mrs. Quick had been receiving relief to the amount of 3s. a week in money and 2s. a week in food. One of the children contracted scarlet fever and was taken to the isolation hospital, and another, a boy of fourteen, had adenoids and was sent to the infirmary. During their absence their relief was stopped. The mother was anxious that the boy with adenoids should complete his school attendance so that he could go to work. Hence, when the boy had been in the infirmary for seventeen days and the operation for removing them had not been done, she brought him home again. On account of this her relief was stopped altogether, and when she asked for help she was given an order for the workhouse. She refused this, for she did not wish to break up her home, and on the face of it it would seem that she is wise. Apart from the fact that it is not good for the children to see the inside of a workhouse if they can be kept outside, it appeared that Mrs. Quick had earned 7s. the previous week by charing; and it is a pity to send to the workhouse a woman who can keep herself, even barely, in the outer world. When a Guardian brought up the case, it was argued against the woman that she had a violent temper, and had once threatened to throw vitriol on a relief officer. On the other hand, she was very ignorant—so uneducated that she had to ask someone to read her relief order—and when she found that it was a workhouse order some indignation may be excused.

### THE POOR LAW'S ADMINISTRATION

We do not wish to take sides against the Guardians, for we know that they must be guided in many cases by their knowledge of the applicant's character. But we certainly think that the reasonable action of the mother in withdrawing her child from the infirmary when an operation which ought to have been done immediately was delayed for more than a fortnight is to be commended. Surely the poorest have the right to protest. The mother's action was not a just ground for stopping the very inadequate relief given for her three children, which she supplemented by her own exertions. We agree with the Guardian who said that "the Poor Law was a good law if well administered, but a Board could make it a bad law." It does not seem, on the face of things, that the Leeds Guardians are administering the law well or humanely. We hope this case may attract sufficient public attention in Leeds to secure that the poorer citizens may in future be guaranteed more just, more courageous, and more generous treatment by this Board.

### MR. MORTON LATHAM, "GUARDIAN OF THE POOR."

Again we have to draw attention to the conduct of the Farnham Board of Guardians, this time not for raising a technical point of some interest, but for arousing the grave misgivings of all humane men. Thanks to the courageous publicity given to the conduct of their chairman by "The Eye-Witness," which deserves great credit for its action, we are able to make more widely known their conduct in refusing to restore to her mother and father a daughter once adopted by the Guardians. No reason was given for this, as the following description of what occurred, which we take from "The Eye-Witness," will show:

"The Chairman (Mr. Morton Latham) told the parents that their request was one that could not be granted.—The Mother: What! Do you think that I am not to see my daughter any more? I brought her up from my own breast, I did.—The Chairman : You are not to have her.—The Mother : What ! Not at all ?—The Chairman : No.—The Mother : What ! Never ?—The Chairman : No.

At any rate it ought to be made clear what led the Guardians originally to adopt the child. On those lines some excuse might be proffered, if excuse there be. If none be given, the voluntary hospital, which has been an important means of humanising the Poor Law infirmary, may here see an example of what modern Guardians are still capable of in that part of their work which the voluntary hospital spirit has been unable to touch or to transform.

### THE WEIR HOSPITAL CHARITY AGAIN.

Its Local Government Committee reports to the London County Council that the scheme prepared by the Charity Commissioners in reference to the Weir Hospital Charity, which has now been sealed, differs in one or two respects from draft schemes previously before the Council. It was proposed by the draft scheme to authorise the trustees, with the approval of the Charity Commissioners, to purchase any property adjoining that in Grove Road, Clapham Park, bequeathed by the testator, so as to enlarge the site of the proposed hospital. Since the draft scheme was framed the Commissioners, we are informed, have authorised the trustees to purchase an adjoining house, and the consequential alterations have been made in the final scheme. The buildings on the two properties have been pulled down, and it is proposed to erect a new cottage hospital on the site. In the draft scheme a note appeared to the effect that the income of the charity was subject to the payment of certain annuities, amounting to £118 4s., bequeathed by the founder of the Charity. The final scheme provides, in addition to these annuities, for certain expenditure in connection with the internal repair and decoration of St. Mary's Church, Balham, the preaching of a sermon by the vicar of the said church, and the defraying of the cost of cleansing and maintaining in repair the founder's mural tablet and memorial. Presumably these provisions are in accordance with the testator's will, but were omitted from the draft scheme. Is this, then, the peaceful and final end of the Weir Charity controversy so often alluded to in these pages, and so slow of decision?

### PUBLIC LAVATORIES IN LONDON.

The visitor to New York is unfavourably impressed by the lack of public conveniences in that city, due, we believe, to the opposition of public-house proprietors who believe that their trade will be interfered with if such conveniences are placed, as they ought to be placed, at every street corner. The visitor to London, no doubt, will be equally unfavourably impressed by the state of the public conveniences in some of our squares. The gentlemen's lavatory in Charing Cross Road is in a condition which urgently calls for the interference of the sanitary inspector. It was, at all events the other day, dirty, ill odorous, and in want of attention. Similarly the lavatories in Tottenham Court Road, at the Elephant and Castle, and in several other quarters need attention. It may not be possible to keep the walls of these places free from pornographic inscriptions, though a great deal may be done towards preventing such disfigurement by providing the walls with coats of some dark coloured paint if black marble tiles cannot be procured; but it should, at all events, be possible to keep urinals and w.c.s clean and sweet by a liberal use of water. The indiscriminate use of disinfectants is to be deprecated since they more often merely disguise the dirt that they cannot effectually remove. More lavatory accommodation is urgently needed in almost every London thoroughfare.

### THE STRAIN OF MENTAL WORK ON WOMEN.

Having touched on recurrent insanity and its cost often of late, it is interesting to note that Cardiff Mental Hospital claims to have its readmissions as low as 8 per cent. The recently disputed "animal house" for research work is to have £370 more spent upon it, Dr. Edwin Goodall, the medical superintendent's last request for the scientific requirements of this scheme. It is worth noting that the matron, Miss Raynes, is to have her annual holiday extended to one month, Dr. Goodall being of opinion that a woman in a mental hospital requires more holidays than a man. Probably most institutional managers will agree with this verdict, and no one can think even now that one month off from such work is excessive.

### YOUNG OUT-PATIENTS AT PLAY.

Thanks to the generosity of some of the hospital's friends, about 200 out-patients of tender years were entertained at the Gartside department of the Manchester Children's Hospital, Pendlebury, last Saturday. One of the Hon. Treasurers, Mr. A. A. Gillies, J.P., Mr. J. Howson Ray, F.R.C.S., Dr. H. R. Hutton, Mr. Charles Roberts, F.R.C.S., Dr. C. Christopher Heywood, Dr. C. P. Lapage, Mr. F. H. Westmacott, F.R.C.S., and other members of the medical and surgical staff were present. The excellent arrangements made and the good taste shown in the decoration of the out-patient hall reflect great credit upon Miss Munroe, the out-patient sister, and her assistants.

### THIS WEEK'S DRUG MARKET.

There has been little if any improvement in business during the week, and the drug market continues very quiet. The most important price movement is the advance of 4s. 6d. per lb. in the quotation for santonin, this being the second advance since the beginning of the year; the price is now three or four times the normal value. Makers of salicylic acid and sodium salicylate have raised their quotations slightly owing to the dearness of carbolic acid. Quicksilver has been advanced in price, but only to a small extent. The high value of opium is firmly maintained. Essence of lemon and essence of bergamot are dearer. Cream of tartar is tending lower in value, as also is menthol. The position of cod-liver oil has not altered appreciably and up to the present the reports of the results of the fishing received from Norway are conflicting.

